# Correction: Dpp dependent Hematopoietic stem cells give rise to Hh dependent blood progenitors in larval lymph gland of *Drosophila*

**DOI:** 10.7554/eLife.51742

**Published:** 2019-09-11

**Authors:** Nidhi Sharma Dey, Parvathy Ramesh, Mayank Chugh, Sudip Mandal, Lolitika Mandal

Dey NS, Ramesh P, Chugh M, Mandal S, Mandal L. 2016. Dpp dependent Hematopoietic stem cells give rise to Hh dependent blood progenitors in larval lymph gland of *Drosophila*. *eLife* **5**:e18295. doi: 10.7554/eLife.18295.

Published 26, October 2016

A reader pointed out that Figure 7R shows same error bars for every sample, while data sheet provided does not suggest so. We presume that while making the final graph for the panel with notation and labeling, somehow we forgot to enter the values for standard deviation for each genotype. By default all the SD bars are identical.

In this connection it is to be noted that there is no error in the original excel file submitted to the journal, linked to the article:

(https://elifesciences.org/articles/18295/figures in the Source data section of the same figure: DOI: http://dx.doi.org/10.7554/eLife.18295.022). The graph plotted in the excel file correctly reflects the data (standard deviation) provided.

The Corrected Figure 7 is shown here:

**Figure:**
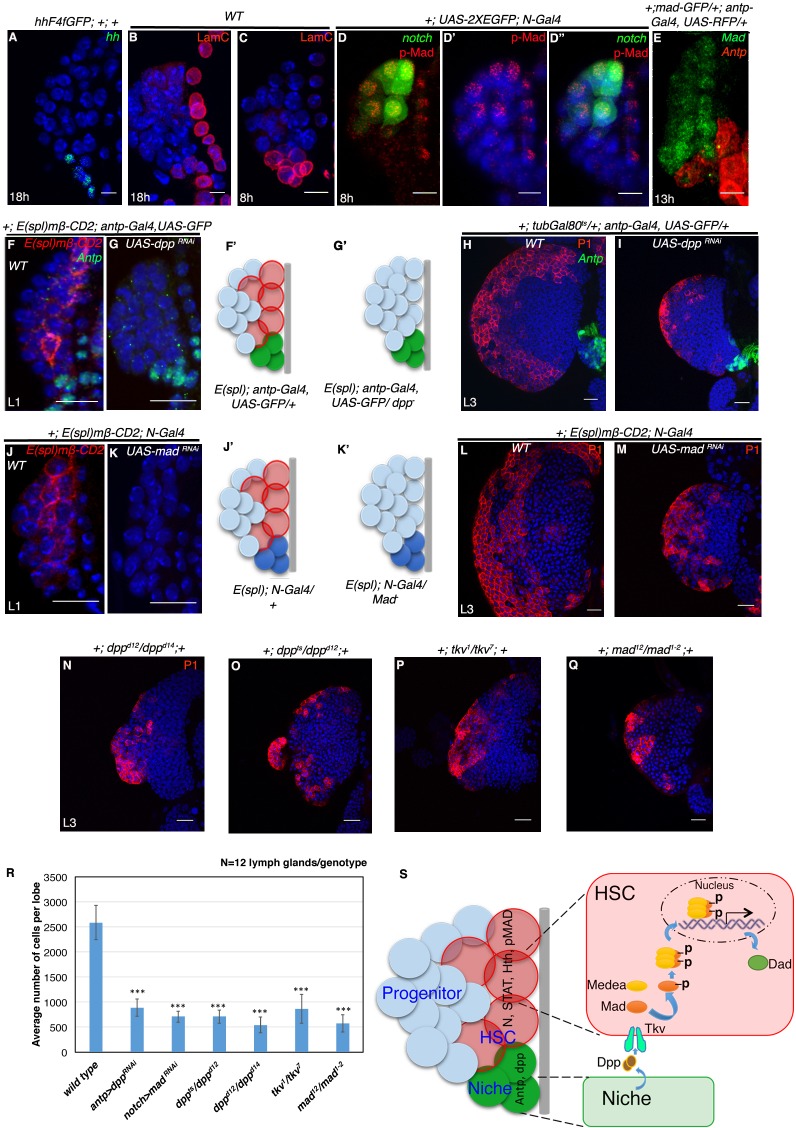


The original published Figure 7 is also shown for reference:

**Figure:**
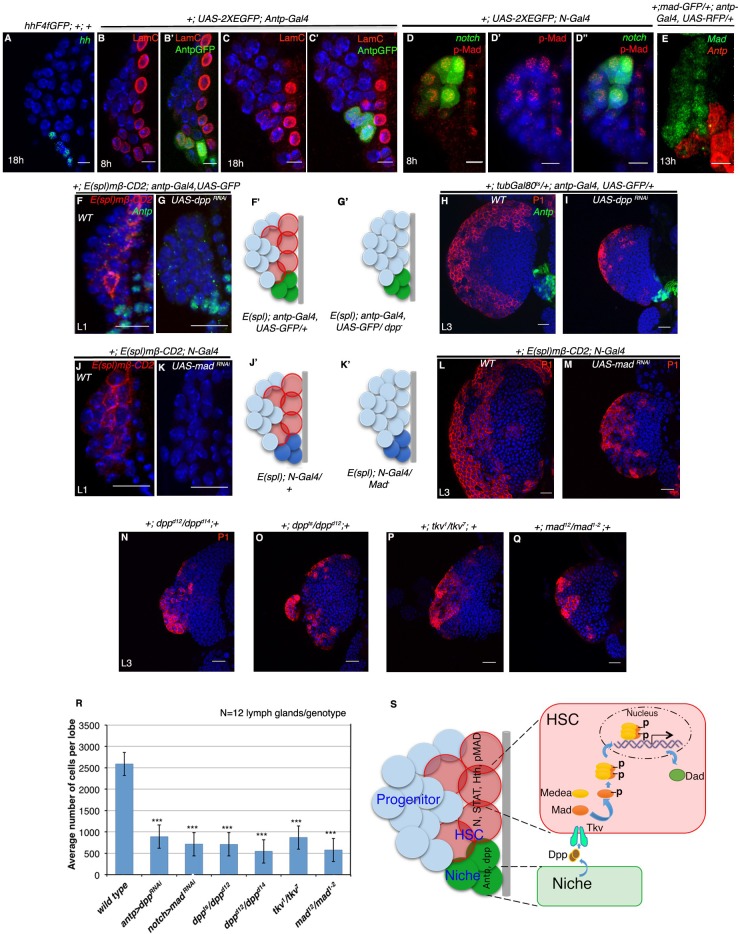


In the process of correcting this error, we thoroughly reviewed the manuscript, and we identified and corrected the following additional error:

The Q panel of Figure 1 has an error in the Graph. The source file that contains both the data and the corresponding graphical representation is correct. However, the graph in the panel Q does not match with that shown in the source file. Therefore, the panel Q of Figure 1 has now been replaced with the one that is in the original source file.

It is to be noted that there is no error in the original excel file submitted to the journal, linked to the article:

**Figure 1—source data 1**

**Contains numerical data plotted in Figure 1Q and R and Total Fluorescence Intensity analyses for Q and R.**

https://doi.org/10.7554/eLife.18295.004

Download elife-18295-fig1-data1-v4.xlsx

The Corrected Figure 1 is shown here:

**Figure:**
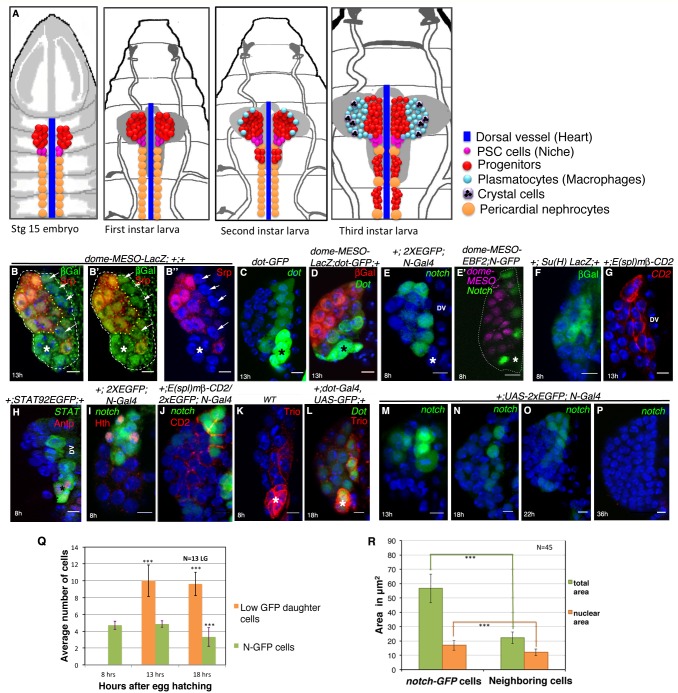


The original published Figure 1 is also shown for reference:

**Figure:**
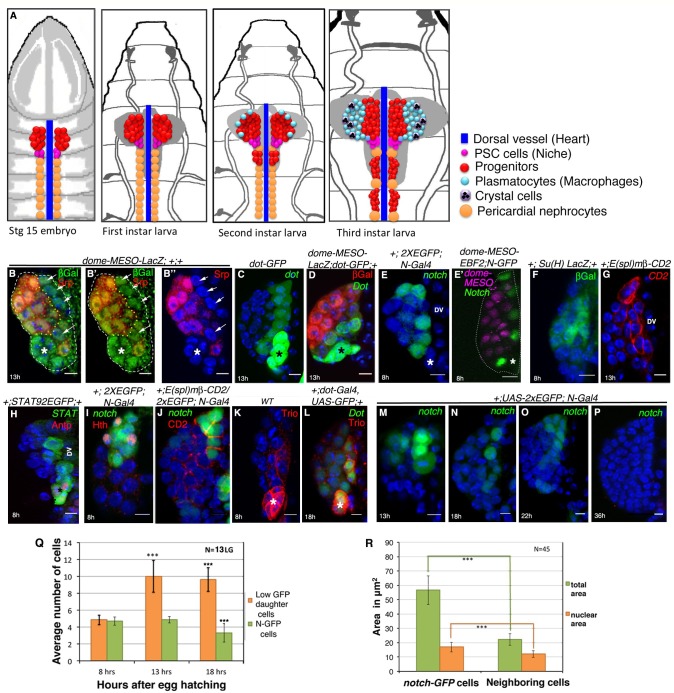


Please note that this correction does not affect the results and conclusions of the original paper. We apologize for the mistake.

